# Di-μ-but-2-enoato-bis­[diaqua­bis(but-2-enoato)neodymium(III)] 2,6-diamino­purine disolvate

**DOI:** 10.1107/S1600536811028558

**Published:** 2011-08-02

**Authors:** Ana María Atria, Alan Astete, Maria Teresa Garland, Ricardo Baggio

**Affiliations:** a Facultad de Ciencias Químicas y Farmacéuticas, Universidad de Chile, Casilla 233, Santiago, Chile; b Departamento de Física, Facultad de Ciencias Físicas y Matemáticas, Universidad de Chile, Santiago de Chile, Chile; c Departamento de Física, Centro Atómico Constituyentes, Comisión Nacional de Energía Atómica, Buenos Aires, Argentina

## Abstract

The title Nd complex [Nd_2_(C_4_H_5_O_2_)_6_(H_2_O)_4_]·2C_5_H_6_N_6_ is isotypic with two previously reported Dy and Ho isologues. It is composed of [Nd(crot)_3_(H_2_O)_2_]_2_ dimers [crot(onate) = but-2-enoate = C_4_H_5_O_2_], built up around symmetry centres and completed by 2,6-diamine­purine mol­ecules acting as solvates. The neodymium cations are coordinated by three chelating crotonato units and two water mol­ecules. One of the chelating carboxyl­ates acts also in a bridging mode, sharing one oxygen with both cations, and the final result is a pair of NdO_9_ tricapped prismatic polyhedra linked to each other through a central (Nd—O)_2_ loop. A most attractive aspect of the structures resides in the existence of a complex inter­molecular hydrogen-bonding interaction scheme involving two sets of tightly inter­linked, non-inter­secting one-dimensional structures, one of them formed by the [Nd(crot)_3_(H_2_O)_2_]_2_ dimers running along [100] and the second by the solvate mol­ecules evolving along [010].

## Related literature

For the Dy and Ho isologues, see: Atria *et al.* (2009 ▶). For hydrogen-bond motifs, see: Bernstein *et al.* (1995 ▶).
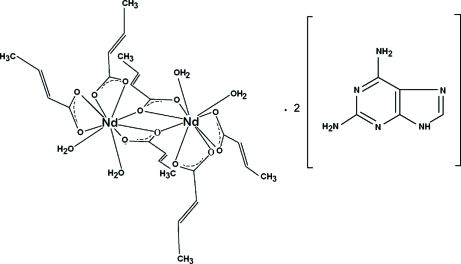


## Experimental

### 

#### Crystal data

[Nd_2_(C_4_H_5_O_2_)_6_(H_2_O)_4_]·2C_5_H_6_N_6_
*M* *_r_* = 1171.34
Triclinic, 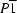

*a* = 8.6441 (2) Å
*b* = 11.1173 (3) Å
*c* = 13.3944 (3) Å
α = 101.230 (9)°
β = 107.522 (11)°
γ = 106.591 (10)°
*V* = 1119.51 (15) Å^3^
*Z* = 1
Mo *K*α radiation
μ = 2.37 mm^−1^
*T* = 150 K
0.24 × 0.20 × 0.14 mm

#### Data collection

Bruker SMART CCD area-detector diffractometer
Absorption correction: multi-scan (*SADABS*; Bruker, 2002 ▶) *T*_min_ = 0.57, *T*_max_ = 0.72
9674 measured reflections
4941 independent reflections
4663 reflections with *I* > 2σ(*I*)
*R*_int_ = 0.011

#### Refinement

*R*[*F* ^2^ > 2σ(*F* ^2^)] = 0.024
*wR*(*F* ^2^) = 0.061
*S* = 1.07
4941 reflections
292 parameters
H-atom parameters constrained
Δρ_max_ = 1.29 e Å^−3^
Δρ_min_ = −0.85 e Å^−3^

### 

Data collection: *SMART-NT* (Bruker, 2001 ▶); cell refinement: *SAINT-NT* (Bruker, 2002 ▶); data reduction: *SAINT-NT*; program(s) used to solve structure: *SHELXS97* (Sheldrick, 2008 ▶); program(s) used to refine structure: *SHELXL97* (Sheldrick, 2008 ▶); molecular graphics: *SHELXTL-NT* (Sheldrick, 2008 ▶); software used to prepare material for publication: *SHELXTL-NT* and *PLATON* (Spek, 2009 ▶).

## Supplementary Material

Crystal structure: contains datablock(s) global, I. DOI: 10.1107/S1600536811028558/bt5580sup1.cif

Structure factors: contains datablock(s) I. DOI: 10.1107/S1600536811028558/bt5580Isup2.hkl

Additional supplementary materials:  crystallographic information; 3D view; checkCIF report

## Figures and Tables

**Table 1:** Hydrogen-bond geometry (Å, °)

*D*—H⋯*A*	*D*—H	H⋯*A*	*D*⋯*A*	*D*—H⋯*A*
N9—H9⋯O22	0.88	1.92	2.784 (3)	167
N6—H6*B*⋯O12^i^	0.88	2.48	3.301 (4)	156
N6—H6*A*⋯N1^ii^	0.88	2.44	3.319 (4)	175
N2—H2*A*⋯N3^iii^	0.88	2.32	3.190 (4)	168
N2—H2*B*⋯O13^iii^	0.88	2.39	3.228 (4)	158
O1*W*—H1*WB*⋯O2*W*^iv^	0.84	2.20	2.959 (3)	150
O1*W*—H1*WA*⋯O11^v^	0.85	1.89	2.699 (3)	158
O2*W*—H2*WB*⋯N7^vi^	0.85	1.81	2.656 (3)	177
O2*W*—H2*WA*⋯O12^iv^	0.84	1.84	2.665 (3)	165
C8—H8⋯O21^vi^	0.95	2.38	3.168 (4)	140
C23—H23⋯N7^vii^	0.95	2.60	3.521 (4)	163
C33—H33⋯N1^iii^	0.95	2.57	3.465 (4)	156

Symmetry codes: (i) 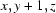; (ii) 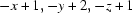; (iii) 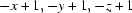; (iv) 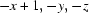; (v) 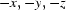; (vi) 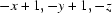; (vii) 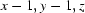.

**Fig. 1.**
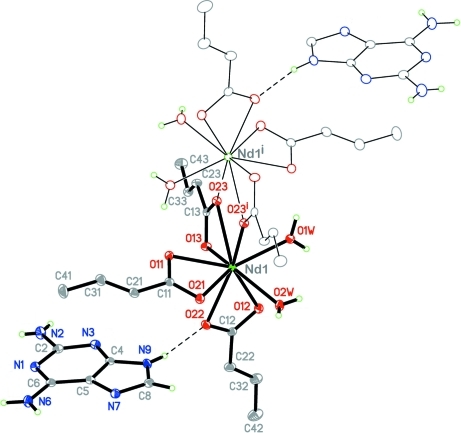
: Ellipsoid plot of (1) drawn at a 40% probability level, with independent (symmetry related) atoms in bold (simple) bonds and filled (empty) ellipsoids. Symmetry codes: (i) -x, -y, -z.

**Fig. 2.**
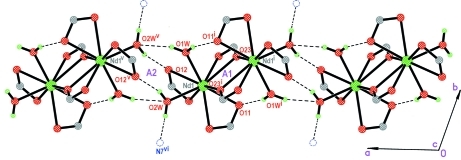
: Packing view of (1) projected down c, showing the 'dimeric' chains and their internal H-bonding linkage (See text). For clarity, only the carboxylato end of the butenoate units have been drawn. Symmetry codes: (i) -x, -y, -z;.(v) -x + 1, -y, -z

**Fig. 3.**
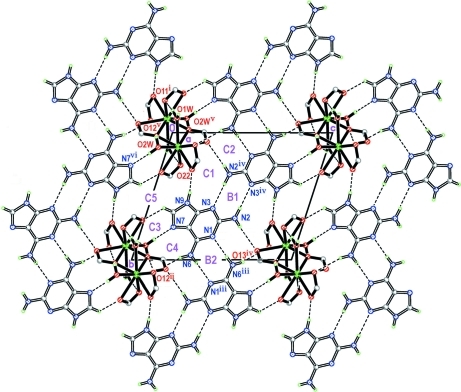
: Packing view of (1) projected down a, showing on the projection plane (running vertically, in hollow bonds) the 'solvato' chains. Perpendicular to the latter and coming out of the plane (in bold, full bonds) the 'dimeric' chains. Intra- as well as inter-chain H-bonds shown in broken lines. For clarity, only the carboxylato end of the butenoate units have been drawn. Symmetry codes: (i) -x, -y, -z; (ii) x, y + 1, z; (iii) -x + 1, -y + 2, -z + 1; (iv) -x + 1, -y + 1, -z + 1; (v) -x + 1, -y, -z; (vi) -x + 1, -y + 1, -z; (vii) x - 1, y - 1, z.

## supplementary crystallographic information

### Comment

The title Nd complex [C_24_H_38_Nd_2_O_16_, 2(C_5_H_6_N_6_)] is isomorphous to two previously reported Dy and Ho isologues (Atria *et al.*, 2009) and its structure does not present any significant difference with the reported ones, for what much of the following discussion has already been made in the above refrenced paper. In the present Nd complex the three crotonate ligands do not depart from expected geometries, the middle double bonds C2n═C3n (n=1,2,3) being distinctly shorter than the remaining two (mean values: <C1n—C2n>: 1.460 (4); <C2n—C3n>: 1.259 (5); <C3n—C4n>: 1.494 (5) Å), with the carboxylate ends presenting a significant resonance, as disclosed by the tight C—O span (1.231 (3)–1.268 (4) Å).

The dap molecule is planar within experimental error; and its overall geometry appears as featureless; the most attractive aspect of the molecule resides in its extreme involvement in H-bonding. In fact, the existence of a large number of efficient H-bonding donors and acceptors in the structure leads to a very complex intermolecular interaction scheme involving two sets of tightly interlinked, non intersecting H-bonded one-dimensional structures, one of them running along the crystallographic a direction and formed by the [Nd(crot)_3_(H_2_O)_2_]_2_ dimers; the second, evolving along b and formed by dap solvato molecules.

Both types of chains embed two sets of inversion centres. In the case of the dimeric chain shown in Fig.2, the same centre (site A1) which relates the two molecules in the dimer through a 4 atoms coordination loop also links them through a *R*_2_^2^(8) H-bonded ring (Bernstein *et al.*, 1995) almost at right angle to the former loop, *viz*., [(O11—Nd1—O1W—H1WA···)_2_] (in what follows, a 2 subindex in a loop formula will indicate duplication by centring and the ··· symbol, a H-bonding interaction). The second centre (site A2) inter-relates neighbouring dimers into chains through two centrosymmetric *R*_2_^2^(8) motives (*viz*., [(O12—Nd1—O2W—H2WA···)_2_; (O2W—Nd1—O1W—H1WB···)_2_ respectively] and two, non centrosymmetric *R*_2_^2^(6) ones flanking the former and involving just one neadimium cation each, namely (O12—Nd1—O1W—H1WB···O2W—H2WA···) and its centrosymmetric analogue.

A much simpler situation arises in the solvato chain (Fig. 3, hollow2 bonds in weak lining), which also contains two independent symmetry centres (noted as B1 and B2), and giving raise to just two, exactly similar centrosymmetric *R*_2_^2^(8) motifs [(N3—C2—N2—H2A···)_2_. and (N1—C6—N6—H6A···)_2_]

The two perpendicular, non intersecting families of chains (the 'dimeric' one, running parallel to [100] at *y*≈*z*≈ 0. and the 'solvato' one, parallel to [010] at *x*≈*z*≈ 1/2) interact at their point of maximal approach through a variety of H-bonds in which there are donors and acceptors at both sides (Fig 3 and Table 1), and which determine four non-centrosymmetric (sites C1, C2, C3 and C4 in Fig. 3) and one centrosymmetric (site C5) H-bonded cycles, with Graph set descriptors *R*_3_^3^(10); *R*_4_^4^(14) (*R*_2_^2^(7), *R*_3_^2^(9) and *R*_4_^4^(14), respectively.

There are also inter-dimeric interactions of the π···π type mediated by symmetry related crotonato double bonds, as in the case between C21═C31 and its (-*x*, 1 - *y*, -*z*) image, characterized by an intercentroid distance of 3.547 (1) Å and a slippage angle of 25.3 (1)°. These interactions link along the [001] direction the dimeric chains which run along [100].

### Experimental

A mixture of Nd_2_O_3_ (1 mmol) and crotonic acid (3 mmol) was dissolved in water (100 mmol),followed by the addition of the 2,6-diaminopurine ligand (1 mmol) dissolved in methanol (10 ml). The resultant mixture was refluxed for 24 h, filtered while hot, and then concentrated to 25 ml. The filtrate was left at room temperature. On standing, colorless crystals suitable for single-crystal X-ray diffraction appeared, which were used without further processing.

### Refinement

All the H atoms were clearly seen in a difference Fourier; they were, however, further idealized at their expected positions and allowed to ride both in coordinates (C—H = 0.93–0.98, N–H = 0.88 Å), as well as in their isotropic displacement factors (*U*_iso_(H) = 1.2/1.5× U_equiv_(host).

### Crystal data

**Table:**

[Nd_2_(C_4_H_5_O_2_)_6_(H_2_O)_4_]·2C_5_H_6_N_6_	*Z* = 1
*M**_r_* = 1171.34	*F*(000) = 586
Triclinic, *P*1	*D*_x_ = 1.737 Mg m^−^^3^
Hall symbol: -P 1	Mo *K*α radiation, λ = 0.71073 Å
*a* = 8.6441 (2) Å	Cell parameters from 4220 reflections
*b* = 11.1173 (3) Å	θ = 1.9–25.7°
*c* = 13.3944 (3) Å	µ = 2.37 mm^−^^1^
α = 101.230 (9)°	*T* = 150 K
β = 107.522 (11)°	Block, colourless
γ = 106.591 (10)°	0.24 × 0.20 × 0.14 mm
*V* = 1119.51 (15) Å^3^	

### Data collection

**Table:**

Bruker SMART CCD area-detector diffractometer	4941 independent reflections
Radiation source: fine-focus sealed tube	4663 reflections with *I* > 2σ(*I*)
graphite	*R*_int_ = 0.011
phi and ω scans	θ_max_ = 28.3°, θ_min_ = 1.7°
Absorption correction: multi-scan (*SADABS*;Bruker, 2002)	*h* = −11→11
*T*_min_ = 0.57, *T*_max_ = 0.72	*k* = −13→14
9674 measured reflections	*l* = −17→17

### Refinement

**Table:**

Refinement on *F*^2^	Primary atom site location: structure-invariant direct methods
Least-squares matrix: full	Secondary atom site location: difference Fourier map
*R*[*F*^2^ > 2σ(*F*^2^)] = 0.024	Hydrogen site location: inferred from neighbouring sites
*wR*(*F*^2^) = 0.061	H-atom parameters constrained
*S* = 1.07	*w* = 1/[σ^2^(*F*_o_^2^) + (0.0304*P*)^2^ + 1.1713*P*] where *P* = (*F*_o_^2^ + 2*F*_c_^2^)/3
4941 reflections	(Δ/σ)_max_ = 0.001
292 parameters	Δρ_max_ = 1.29 e Å^−^^3^
0 restraints	Δρ_min_ = −0.85 e Å^−^^3^

### Special details

**Table:**

Geometry. All e.s.d.'s (except the e.s.d. in the dihedral angle between two l.s. planes) are estimated using the full covariance matrix. The cell e.s.d.'s are taken into account individually in the estimation of e.s.d.'s in distances, angles and torsion angles; correlations between e.s.d.'s in cell parameters are only used when they are defined by crystal symmetry. An approximate (isotropic) treatment of cell e.s.d.'s is used for estimating e.s.d.'s involving l.s. planes.

### Fractional atomic coordinates and isotropic or equivalent isotropic displacement parameters (Å^2^)

**Table:**

	*x*	*y*	*z*	*U*_iso_*/*U*_eq_	
Nd1	0.260333 (17)	0.113788 (13)	0.062493 (11)	0.02382 (5)	
O11	0.1168 (3)	0.2601 (2)	0.11102 (18)	0.0352 (4)	
O21	0.2709 (3)	0.3212 (2)	0.01582 (17)	0.0359 (5)	
C11	0.1849 (4)	0.3454 (3)	0.0708 (2)	0.0305 (6)	
C21	0.1654 (4)	0.4734 (3)	0.0913 (3)	0.0398 (7)	
H21	0.2010	0.5305	0.0514	0.048*	
C31	0.1017 (5)	0.5111 (4)	0.1615 (3)	0.0495 (9)	
H31	0.0616	0.4502	0.1974	0.059*	
C41	0.0853 (6)	0.6405 (4)	0.1915 (4)	0.0722 (13)	
H41A	0.1315	0.6946	0.1499	0.108*	
H41B	0.1513	0.6850	0.2705	0.108*	
H41C	−0.0379	0.6278	0.1741	0.108*	
O12	0.5504 (3)	0.1008 (2)	0.16470 (18)	0.0385 (5)	
O22	0.5282 (3)	0.2922 (2)	0.19938 (18)	0.0376 (5)	
C12	0.6183 (4)	0.2221 (3)	0.2132 (2)	0.0326 (6)	
C22	0.8009 (5)	0.2872 (5)	0.2872 (3)	0.0562 (10)	
H22	0.8379	0.3779	0.3263	0.067*	
C32	0.9095 (6)	0.2381 (5)	0.3037 (4)	0.0692 (12)	
H32	0.8756	0.1472	0.2663	0.083*	
C42	1.0982 (6)	0.3138 (8)	0.3813 (5)	0.116 (3)	
H42A	1.1107	0.4015	0.4223	0.174*	
H42B	1.1731	0.3228	0.3388	0.174*	
H42C	1.1328	0.2659	0.4330	0.174*	
O13	0.2514 (3)	0.0698 (2)	0.23400 (17)	0.0367 (5)	
O23	0.0035 (2)	−0.02354 (19)	0.09761 (15)	0.0305 (4)	
C13	0.0973 (3)	−0.0041 (3)	0.1969 (2)	0.0271 (5)	
C23	0.0184 (4)	−0.0740 (3)	0.2616 (2)	0.0352 (6)	
H23	−0.1026	−0.1264	0.2294	0.042*	
C33	0.1062 (4)	−0.0674 (3)	0.3606 (3)	0.0392 (7)	
H33	0.2243	−0.0081	0.3937	0.047*	
C43	0.0379 (5)	−0.1449 (4)	0.4274 (3)	0.0576 (10)	
H43A	−0.0858	−0.1991	0.3856	0.086*	
H43B	0.0512	−0.0845	0.4960	0.086*	
H43C	0.1034	−0.2020	0.4444	0.086*	
O1W	0.2282 (3)	−0.1192 (2)	−0.00386 (19)	0.0403 (5)	
H1WA	0.1293	−0.1771	−0.0462	0.048*	
H1WB	0.3099	−0.1466	−0.0023	0.048*	
O2W	0.4103 (3)	0.1137 (2)	−0.06425 (18)	0.0369 (5)	
H2WA	0.4049	0.0384	−0.0961	0.044*	
H2WB	0.4146	0.1621	−0.1056	0.044*	
N1	0.5071 (3)	0.8157 (2)	0.4590 (2)	0.0354 (5)	
C2	0.5012 (4)	0.6968 (3)	0.4716 (2)	0.0352 (6)	
N2	0.4739 (4)	0.6752 (3)	0.5609 (2)	0.0506 (7)	
H2A	0.4928	0.6065	0.5776	0.061*	
H2B	0.5222	0.7482	0.6167	0.061*	
N3	0.5160 (3)	0.5994 (2)	0.4061 (2)	0.0338 (5)	
C4	0.5414 (4)	0.6308 (3)	0.3204 (2)	0.0300 (6)	
C5	0.5486 (4)	0.7467 (3)	0.2965 (2)	0.0304 (6)	
C6	0.5307 (4)	0.8426 (3)	0.3711 (2)	0.0319 (6)	
N6	0.5395 (4)	0.9612 (3)	0.3599 (2)	0.0425 (6)	
H6A	0.5220	1.0164	0.4081	0.051*	
H6B	0.5347	0.9734	0.2962	0.051*	
N7	0.5774 (3)	0.7426 (2)	0.2006 (2)	0.0359 (5)	
C8	0.5872 (4)	0.6272 (3)	0.1702 (3)	0.0373 (7)	
H8	0.6058	0.5959	0.1052	0.045*	
N9	0.5685 (3)	0.5568 (2)	0.2398 (2)	0.0345 (5)	
H9	0.5731	0.4778	0.2338	0.041*	

### Atomic displacement parameters (Å^2^)

**Table:**

	*U*^11^	*U*^22^	*U*^33^	*U*^12^	*U*^13^	*U*^23^
Nd1	0.02283 (8)	0.02416 (8)	0.02502 (8)	0.00972 (6)	0.00944 (6)	0.00655 (6)
O11	0.0376 (11)	0.0296 (10)	0.0449 (12)	0.0148 (9)	0.0221 (10)	0.0113 (9)
O21	0.0419 (12)	0.0363 (11)	0.0393 (11)	0.0197 (9)	0.0209 (10)	0.0157 (9)
C11	0.0264 (13)	0.0292 (14)	0.0300 (14)	0.0112 (11)	0.0047 (11)	0.0044 (11)
C21	0.0367 (16)	0.0324 (16)	0.0485 (18)	0.0168 (13)	0.0110 (14)	0.0110 (14)
C31	0.048 (2)	0.0411 (19)	0.052 (2)	0.0232 (16)	0.0112 (16)	0.0028 (16)
C41	0.071 (3)	0.058 (3)	0.078 (3)	0.043 (2)	0.012 (2)	−0.006 (2)
O12	0.0328 (11)	0.0375 (12)	0.0443 (12)	0.0196 (9)	0.0105 (9)	0.0073 (10)
O22	0.0350 (11)	0.0269 (10)	0.0421 (12)	0.0099 (9)	0.0076 (9)	0.0048 (9)
C12	0.0302 (14)	0.0379 (16)	0.0277 (14)	0.0093 (12)	0.0122 (11)	0.0088 (12)
C22	0.0336 (18)	0.075 (3)	0.050 (2)	0.0154 (18)	0.0088 (16)	0.0157 (19)
C32	0.052 (2)	0.088 (3)	0.064 (3)	0.025 (2)	0.018 (2)	0.023 (2)
C42	0.039 (2)	0.206 (8)	0.091 (4)	0.027 (3)	0.008 (2)	0.074 (5)
O13	0.0275 (10)	0.0437 (12)	0.0300 (10)	0.0035 (9)	0.0071 (8)	0.0129 (9)
O23	0.0285 (10)	0.0339 (10)	0.0267 (10)	0.0101 (8)	0.0081 (8)	0.0098 (8)
C13	0.0290 (13)	0.0274 (13)	0.0267 (13)	0.0125 (11)	0.0114 (11)	0.0079 (11)
C23	0.0292 (14)	0.0385 (16)	0.0360 (15)	0.0079 (12)	0.0128 (12)	0.0135 (13)
C33	0.0384 (16)	0.0445 (18)	0.0335 (16)	0.0115 (14)	0.0151 (13)	0.0127 (14)
C43	0.062 (2)	0.071 (3)	0.045 (2)	0.019 (2)	0.0251 (18)	0.0310 (19)
O1W	0.0308 (11)	0.0298 (11)	0.0576 (14)	0.0131 (9)	0.0163 (10)	0.0052 (10)
O2W	0.0533 (13)	0.0362 (11)	0.0409 (12)	0.0258 (10)	0.0301 (10)	0.0202 (9)
N1	0.0407 (14)	0.0333 (13)	0.0329 (13)	0.0153 (11)	0.0132 (11)	0.0108 (10)
C2	0.0371 (15)	0.0354 (15)	0.0302 (14)	0.0103 (13)	0.0112 (12)	0.0113 (12)
N2	0.076 (2)	0.0489 (17)	0.0386 (15)	0.0263 (16)	0.0296 (15)	0.0204 (13)
N3	0.0387 (13)	0.0323 (13)	0.0314 (12)	0.0118 (11)	0.0132 (11)	0.0141 (10)
C4	0.0281 (13)	0.0278 (14)	0.0297 (14)	0.0076 (11)	0.0087 (11)	0.0074 (11)
C5	0.0295 (13)	0.0283 (14)	0.0311 (14)	0.0077 (11)	0.0103 (11)	0.0107 (11)
C6	0.0303 (14)	0.0289 (14)	0.0343 (15)	0.0098 (11)	0.0097 (12)	0.0107 (12)
N6	0.0630 (18)	0.0351 (14)	0.0426 (15)	0.0268 (13)	0.0256 (14)	0.0179 (12)
N7	0.0424 (14)	0.0322 (13)	0.0364 (13)	0.0124 (11)	0.0186 (11)	0.0137 (11)
C8	0.0430 (17)	0.0349 (16)	0.0358 (16)	0.0118 (13)	0.0201 (13)	0.0104 (13)
N9	0.0401 (13)	0.0293 (12)	0.0363 (13)	0.0136 (11)	0.0162 (11)	0.0106 (10)

### Geometric parameters (Å, °)

**Table:**

Nd1—O23^i^	2.3912 (18)	O23—Nd1^i^	2.3912 (18)
Nd1—O11	2.415 (2)	C13—C23	1.461 (4)
Nd1—O2W	2.426 (2)	C23—C33	1.290 (4)
Nd1—O13	2.459 (2)	C23—H23	0.9500
Nd1—O22	2.462 (2)	C33—C43	1.489 (5)
Nd1—O1W	2.474 (2)	C33—H33	0.9500
Nd1—O21	2.489 (2)	C43—H43A	0.9800
Nd1—O12	2.530 (2)	C43—H43B	0.9800
Nd1—O23	2.5402 (19)	C43—H43C	0.9800
O11—C11	1.268 (4)	O1W—H1WA	0.8474
O21—C11	1.238 (3)	O1W—H1WB	0.8415
C11—C21	1.463 (4)	O2W—H2WA	0.8435
C21—C31	1.286 (5)	O2W—H2WB	0.8453
C21—H21	0.9500	N1—C6	1.328 (4)
C31—C41	1.475 (5)	N1—C2	1.353 (4)
C31—H31	0.9500	C2—N3	1.317 (4)
C41—H41A	0.9800	C2—N2	1.339 (4)
C41—H41B	0.9800	N2—H2A	0.8800
C41—H41C	0.9800	N2—H2B	0.8800
O12—C12	1.250 (4)	N3—C4	1.323 (4)
O22—C12	1.250 (4)	C4—N9	1.346 (4)
C12—C22	1.457 (4)	C4—C5	1.376 (4)
C22—C32	1.202 (6)	C5—N7	1.374 (4)
C22—H22	0.9500	C5—C6	1.390 (4)
C32—C42	1.518 (6)	C6—N6	1.339 (4)
C32—H32	0.9500	N6—H6A	0.8800
C42—H42A	0.9800	N6—H6B	0.8800
C42—H42B	0.9800	N7—C8	1.303 (4)
C42—H42C	0.9800	C8—N9	1.346 (4)
O13—C13	1.231 (3)	C8—H8	0.9500
O23—C13	1.269 (3)	N9—H9	0.8800
			
O23^i^—Nd1—O11	80.44 (7)	C22—C32—C42	123.4 (6)
O23^i^—Nd1—O2W	86.10 (7)	C22—C32—H32	118.3
O11—Nd1—O2W	128.71 (7)	C42—C32—H32	118.3
O23^i^—Nd1—O13	118.74 (6)	C32—C42—H42A	109.5
O11—Nd1—O13	81.64 (7)	C32—C42—H42B	109.5
O2W—Nd1—O13	145.49 (7)	H42A—C42—H42B	109.5
O23^i^—Nd1—O22	154.36 (7)	C32—C42—H42C	109.5
O11—Nd1—O22	84.47 (7)	H42A—C42—H42C	109.5
O2W—Nd1—O22	87.34 (8)	H42B—C42—H42C	109.5
O13—Nd1—O22	78.97 (7)	C13—O13—Nd1	97.38 (17)
O23^i^—Nd1—O1W	77.04 (7)	C13—O23—Nd1^i^	155.29 (18)
O11—Nd1—O1W	145.13 (7)	C13—O23—Nd1	92.44 (16)
O2W—Nd1—O1W	75.96 (7)	Nd1^i^—O23—Nd1	112.26 (7)
O13—Nd1—O1W	86.25 (8)	O13—C13—O23	119.0 (3)
O22—Nd1—O1W	125.07 (7)	O13—C13—C23	122.6 (3)
O23^i^—Nd1—O21	82.11 (7)	O23—C13—C23	118.4 (2)
O11—Nd1—O21	52.48 (7)	C33—C23—C13	122.7 (3)
O2W—Nd1—O21	76.82 (7)	C33—C23—H23	118.6
O13—Nd1—O21	126.93 (7)	C13—C23—H23	118.6
O22—Nd1—O21	72.27 (7)	C23—C33—C43	125.5 (3)
O1W—Nd1—O21	146.56 (8)	C23—C33—H33	117.3
O23^i^—Nd1—O12	147.82 (7)	C43—C33—H33	117.3
O11—Nd1—O12	131.72 (7)	C33—C43—H43A	109.5
O2W—Nd1—O12	74.11 (7)	C33—C43—H43B	109.5
O13—Nd1—O12	72.59 (7)	H43A—C43—H43B	109.5
O22—Nd1—O12	51.36 (7)	C33—C43—H43C	109.5
O1W—Nd1—O12	73.72 (7)	H43A—C43—H43C	109.5
O21—Nd1—O12	116.47 (7)	H43B—C43—H43C	109.5
O23^i^—Nd1—O23	67.74 (7)	Nd1—O1W—H1WA	120.1
O11—Nd1—O23	73.71 (7)	Nd1—O1W—H1WB	125.9
O2W—Nd1—O23	143.16 (7)	H1WA—O1W—H1WB	112.2
O13—Nd1—O23	51.01 (6)	Nd1—O2W—H2WA	114.2
O22—Nd1—O23	127.16 (7)	Nd1—O2W—H2WB	125.8
O1W—Nd1—O23	73.26 (7)	H2WA—O2W—H2WB	110.5
O21—Nd1—O23	121.93 (6)	C6—N1—C2	118.7 (3)
O12—Nd1—O23	115.04 (7)	N3—C2—N2	116.3 (3)
C11—O11—Nd1	94.84 (16)	N3—C2—N1	128.0 (3)
C11—O21—Nd1	92.12 (17)	N2—C2—N1	115.7 (3)
O21—C11—O11	119.9 (3)	C2—N2—H2A	116.1
O21—C11—C21	119.9 (3)	C2—N2—H2B	111.5
O11—C11—C21	120.1 (3)	H2A—N2—H2B	114.5
C31—C21—C11	122.2 (3)	C2—N3—C4	111.3 (3)
C31—C21—H21	118.9	N3—C4—N9	127.0 (3)
C11—C21—H21	118.9	N3—C4—C5	127.0 (3)
C21—C31—C41	125.9 (4)	N9—C4—C5	105.9 (3)
C21—C31—H31	117.0	N7—C5—C4	109.9 (3)
C41—C31—H31	117.0	N7—C5—C6	133.2 (3)
C31—C41—H41A	109.5	C4—C5—C6	116.8 (3)
C31—C41—H41B	109.5	N1—C6—N6	118.6 (3)
H41A—C41—H41B	109.5	N1—C6—C5	118.1 (3)
C31—C41—H41C	109.5	N6—C6—C5	123.3 (3)
H41A—C41—H41C	109.5	C6—N6—H6A	119.2
H41B—C41—H41C	109.5	C6—N6—H6B	118.3
C12—O12—Nd1	92.73 (17)	H6A—N6—H6B	120.5
C12—O22—Nd1	95.96 (17)	C8—N7—C5	104.0 (2)
O22—C12—O12	119.8 (3)	N7—C8—N9	113.4 (3)
O22—C12—C22	117.6 (3)	N7—C8—H8	123.3
O12—C12—C22	122.6 (3)	N9—C8—H8	123.3
C32—C22—C12	126.7 (5)	C8—N9—C4	106.7 (3)
C32—C22—H22	116.6	C8—N9—H9	126.7
C12—C22—H22	116.6	C4—N9—H9	126.7
			
O23^i^—Nd1—O11—C11	−91.82 (17)	O21—Nd1—O13—C13	106.38 (18)
O2W—Nd1—O11—C11	−14.7 (2)	O12—Nd1—O13—C13	−143.22 (19)
O13—Nd1—O11—C11	147.00 (17)	O23—Nd1—O13—C13	2.32 (16)
O22—Nd1—O11—C11	67.38 (17)	O23^i^—Nd1—O23—C13	179.2 (2)
O1W—Nd1—O11—C11	−142.05 (17)	O11—Nd1—O23—C13	−94.62 (16)
O21—Nd1—O11—C11	−4.47 (15)	O2W—Nd1—O23—C13	131.21 (16)
O12—Nd1—O11—C11	89.43 (18)	O13—Nd1—O23—C13	−2.24 (15)
O23—Nd1—O11—C11	−161.28 (18)	O22—Nd1—O23—C13	−24.95 (18)
O23^i^—Nd1—O21—C11	88.54 (17)	O1W—Nd1—O23—C13	96.72 (16)
O11—Nd1—O21—C11	4.57 (16)	O21—Nd1—O23—C13	−116.22 (16)
O2W—Nd1—O21—C11	176.36 (18)	O12—Nd1—O23—C13	34.34 (17)
O13—Nd1—O21—C11	−31.7 (2)	O23^i^—Nd1—O23—Nd1^i^	0.0
O22—Nd1—O21—C11	−92.21 (17)	O11—Nd1—O23—Nd1^i^	86.13 (9)
O1W—Nd1—O21—C11	140.16 (17)	O2W—Nd1—O23—Nd1^i^	−48.03 (14)
O12—Nd1—O21—C11	−119.15 (17)	O13—Nd1—O23—Nd1^i^	178.52 (13)
O23—Nd1—O21—C11	31.01 (19)	O22—Nd1—O23—Nd1^i^	155.80 (7)
Nd1—O21—C11—O11	−8.0 (3)	O1W—Nd1—O23—Nd1^i^	−82.53 (9)
Nd1—O21—C11—C21	170.7 (2)	O21—Nd1—O23—Nd1^i^	64.54 (10)
Nd1—O11—C11—O21	8.2 (3)	O12—Nd1—O23—Nd1^i^	−144.91 (8)
Nd1—O11—C11—C21	−170.4 (2)	Nd1—O13—C13—O23	−4.1 (3)
O21—C11—C21—C31	−168.8 (3)	Nd1—O13—C13—C23	174.0 (2)
O11—C11—C21—C31	9.8 (5)	Nd1^i^—O23—C13—O13	−177.7 (3)
C11—C21—C31—C41	176.8 (3)	Nd1—O23—C13—O13	4.0 (3)
O23^i^—Nd1—O12—C12	151.90 (16)	Nd1^i^—O23—C13—C23	4.1 (6)
O11—Nd1—O12—C12	−30.4 (2)	Nd1—O23—C13—C23	−174.3 (2)
O2W—Nd1—O12—C12	97.70 (18)	O13—C13—C23—C33	−2.9 (5)
O13—Nd1—O12—C12	−91.49 (18)	O23—C13—C23—C33	175.2 (3)
O22—Nd1—O12—C12	−1.83 (16)	C13—C23—C33—C43	−174.3 (3)
O1W—Nd1—O12—C12	177.35 (19)	C6—N1—C2—N3	−0.1 (5)
O21—Nd1—O12—C12	31.69 (19)	C6—N1—C2—N2	−178.9 (3)
O23—Nd1—O12—C12	−120.53 (17)	N2—C2—N3—C4	179.6 (3)
O23^i^—Nd1—O22—C12	−145.16 (18)	N1—C2—N3—C4	0.8 (5)
O11—Nd1—O22—C12	160.82 (18)	C2—N3—C4—N9	177.3 (3)
O2W—Nd1—O22—C12	−69.88 (18)	C2—N3—C4—C5	−1.5 (4)
O13—Nd1—O22—C12	78.28 (18)	N3—C4—C5—N7	179.9 (3)
O1W—Nd1—O22—C12	0.9 (2)	N9—C4—C5—N7	0.9 (3)
O21—Nd1—O22—C12	−146.88 (19)	N3—C4—C5—C6	1.4 (4)
O12—Nd1—O22—C12	1.84 (16)	N9—C4—C5—C6	−177.6 (3)
O23—Nd1—O22—C12	96.09 (18)	C2—N1—C6—N6	−178.8 (3)
Nd1—O22—C12—O12	−3.4 (3)	C2—N1—C6—C5	−0.2 (4)
Nd1—O22—C12—C22	176.9 (3)	N7—C5—C6—N1	−178.5 (3)
Nd1—O12—C12—O22	3.3 (3)	C4—C5—C6—N1	−0.4 (4)
Nd1—O12—C12—C22	−177.1 (3)	N7—C5—C6—N6	0.1 (5)
O22—C12—C22—C32	−173.8 (4)	C4—C5—C6—N6	178.2 (3)
O12—C12—C22—C32	6.5 (6)	C4—C5—N7—C8	−0.2 (3)
C12—C22—C32—C42	179.2 (4)	C6—C5—N7—C8	178.0 (3)
O23^i^—Nd1—O13—C13	3.9 (2)	C5—N7—C8—N9	−0.6 (4)
O11—Nd1—O13—C13	78.10 (18)	N7—C8—N9—C4	1.1 (4)
O2W—Nd1—O13—C13	−127.48 (18)	N3—C4—N9—C8	179.8 (3)
O22—Nd1—O13—C13	164.05 (19)	C5—C4—N9—C8	−1.2 (3)
O1W—Nd1—O13—C13	−69.12 (18)		

Symmetry codes: (i) −*x*, −*y*, −*z*.

### Hydrogen-bond geometry (Å, °)

**Table:**

*D*—H···*A*	*D*—H	H···*A*	*D*···*A*	*D*—H···*A*
N9—H9···O22	0.88	1.92	2.784 (3)	167.
N6—H6B···O12^ii^	0.88	2.48	3.301 (4)	156.
N6—H6A···N1^iii^	0.88	2.44	3.319 (4)	175.
N2—H2A···N3^iv^	0.88	2.32	3.190 (4)	168.
N2—H2B···O13^iv^	0.88	2.39	3.228 (4)	158.
O1W—H1WB···O2W^v^	0.84	2.20	2.959 (3)	150.
O1W—H1WA···O11^i^	0.85	1.89	2.699 (3)	158.
O2W—H2WB···N7^vi^	0.85	1.81	2.656 (3)	177.
O2W—H2WA···O12^v^	0.84	1.84	2.665 (3)	165.
C8—H8···O21^vi^	0.95	2.38	3.168 (4)	140.
C23—H23···N7^vii^	0.95	2.60	3.521 (4)	163.
C33—H33···N1^iv^	0.95	2.57	3.465 (4)	156.

Symmetry codes: (ii) *x*, *y*+1, *z*; (iii) −*x*+1, −*y*+2, −*z*+1; (iv) −*x*+1, −*y*+1, −*z*+1; (v) −*x*+1, −*y*, −*z*; (i) −*x*, −*y*, −*z*; (vi) −*x*+1, −*y*+1, −*z*; (vii) *x*−1, *y*−1, *z*.
